# The Properties of the Transient Outward, Inward Rectifier and Acetylcholine-Sensitive Potassium Currents in Atrial Myocytes from Dogs in Sinus Rhythm and Experimentally Induced Atrial Fibrillation Dog Models

**DOI:** 10.3390/ph17091138

**Published:** 2024-08-29

**Authors:** Zsófia Kohajda, Claudia Corici, Attila Kristóf, László Virág, Zoltán Husti, István Baczkó, László Sághy, András Varró, Norbert Jost

**Affiliations:** 1 HUN-REN-SZTE Research Group of Cardiovascular Pharmacology, H-6701 Szeged, Hungary; 2 Department of Pharmacology & Pharmacotherapy, Albert Szent-Györgyi Medical School, University of Szeged, Dóm tér 12, P.O. Box 427, H-6701 Szeged, Hungarybaczko.istvan@med.u-szeged.hu (I.B.); 3 Pharmaceutical and Medical Device Developments Competence Centre of the Life Sciences Cluster, Centre of Excellence for Interdisciplinary Research, Development and Innovation, University of Szeged, H-6701 Szeged, Hungary; 4 Cardiac Electrophysiology Division, Department of Internal Medicine, Albert Szent-Györgyi Medical School, University of Szeged, H-6701 Szeged, Hungary

**Keywords:** antiarrhythmic drugs, atrial fibrillation-induced remodeling, cardiac electrophysiology, acetylcholine-sensitive potassium current, proarrhythmia

## Abstract

Aims: Atrial fibrillation (AF) is the most common chronic/recurrent arrhythmia, which significantly impairs quality of life and increases cardiovascular morbidity and mortality. Therefore, the aim of the present study was to investigate the properties of three repolarizing potassium currents which were shown to contribute to AF-induced electrical remodeling, i.e., the transient outward (I_to_), inward rectifier (I_K1_) and acetylcholine-sensitive (I_K,ACh_) potassium currents in isolated atrial myocytes obtained from dogs either with sinus rhythm (SR) or following chronic atrial tachypacing (400/min)-induced AF. Methods: Atrial remodeling and AF were induced by chronic (4–6 weeks of) right atrial tachypacing (400/min) in dogs. Transmembrane ionic currents were measured by applying the whole-cell patch-clamp technique at 37 °C. Results: The I_to_ current was slightly downregulated in AF cells when compared with that recorded in SR cells. This downregulation was also associated with slowed inactivation kinetics. The I_K1_ current was found to be larger in AF cells; however, this upregulation was not statistically significant in the voltage range corresponding with atrial action potential (−80 mV to 0 mV). I_K,ACh_ was activated by the cholinergic agonist carbachol (CCh; 2 µM). In SR, CCh activated a large current either in inward or outward directions. The selective I_K,ACh_ inhibitor tertiapin (10 nM) blocked the outward CCh-induced current by 61%. In atrial cardiomyocytes isolated from dogs with AF, the presence of a constitutively active I_K,ACh_ was observed, blocked by 59% with 10 nM tertiapin. However, in “AF atrial myocytes”, CCh activated an additional, significant ligand-dependent and tertiapin-sensitive I_K,ACh_ current. Conclusions: In our dog AF model, I_to_ unlike in humans was downregulated only in a slight manner. Due to its slow inactivation kinetics, it seems that I_to_ may play a more significant role in atrial repolarization than in ventricular working muscle myocytes. The presence of the constitutively active I_K,ACh_ in atrial myocytes from AF dogs shows that electrical remodeling truly developed in this model. The I_K,ACh_ current (both ligand-dependent and constitutively active) seems to play a significant role in canine atrial electrical remodeling and may be a promising atrial selective drug target for suppressing AF.

## 1. Introduction

Atrial fibrillation (AF) is the most common recurrent arrhythmia in clinical practice, largely affecting the quality of life. AF is characterized by disorganized, high-rate (up to 300–600/min) atrial electrical activity and it is associated with shorter action potential durations (APDs), effective refractory periods (ERPs) and a loss of rate-dependent APD adaptation that involves concomitant alterations in ion current activity [1,2]. When AF turns to permanent form, lifelong pharmacological treatments or surgical interventions (electrical cardioversion) are necessary. However, pharmacological treatments often have the risks of proarrhythmic effects, so the development of atrial-specific antiarrhythmic drugs has become a central interest of AF research. It is well known that in long-time (persistent or permanent) forms of AF, atrial remodeling develops at electrophysiological, contractile and structural levels [1,2,3,4,5]. These changes in the atria promote the persistence, recurrence and perpetuation of AF. 

Atrial fibrillation-induced structural remodeling (especially fibrosis) causes stiffness, which inhibits proper atrial loading [1,3,4,5]. Atrial tachycardia associated with electrophysiological atrial remodeling induces several atrial ion channel functional alterations [1,2,4,5,6]. Typically, an abbreviation of the APD (up to 30%) and the triangularization of action potentials (APs) can be observed [6]. This phenomenon could be based on the AF-induced downregulation of I_CaL_, which alters the myocardial Ca^2+^ homeostasis [7,8]. Furthermore, the AF-induced upregulation of the inward rectifier (I_K1_), activation of the constitutively active G-protein gated acetylcholine sensitive (I_KACh_) and downregulation of the transient outward potassium current (I_to_) strengthen this process [7,9,10]. The alteration of other transmembrane currents (I_Na_, I_Kur_, I_NCX_, etc.) in the permanent forms of AF was reported; however, the contribution of these currents seemed to not be so important to the above-mentioned AF-induced AP remodeling [1,2,5,6]. These transmembrane ion channel changes have also been intensively investigated in human atrial samples, but regarding their importance from the point of view of clinical drug development, the investigation of AF in different animal models is required. 

In this study, we set up and developed a tachypaced dog model of AF, to investigate the possible changes in different potassium channels—including I_to_, I_K1_ and I_K,Ach_—which, based on previous reports, are considered to be the main pharmacological targets in AF treatments [1,2].

## 2. Results

### 2.1. The Transient Outward Potassium Current (I_to_)

**Figure 1:**
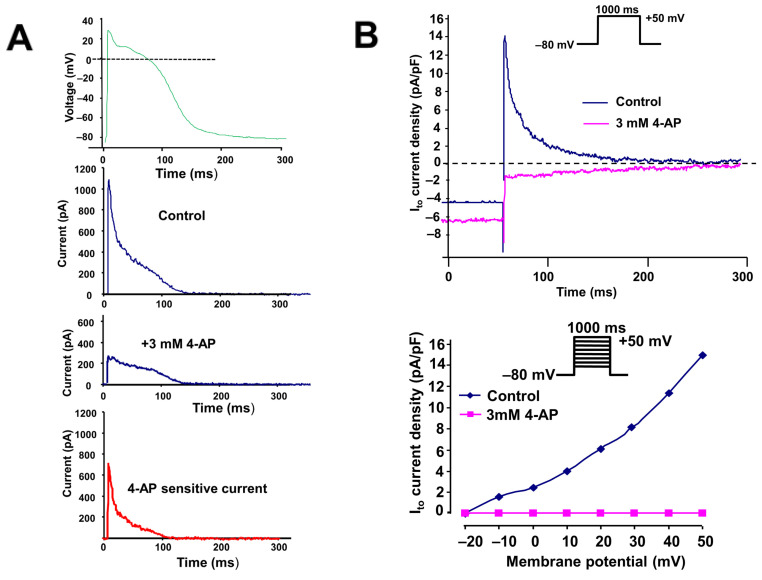
(**A**) Identification of a 3 mM 4-aminopyridine (4-AP)-sensitive transient outward potassium current (I_to_) using an action potential-like voltage pulse in an isolated dog (in SR) right atrial myocyte. The upper panel indicates the applied voltage pulse. (**B**) Original transient outward potassium current traces activated by rectangular voltages before and after application of 3 µM 4-AP and rectangle voltage pulse in an isolated dog (in SR) right atrial myocyte (top), and the corresponding current–voltage (I–V) relationship (bottom).

**Figure 2:**
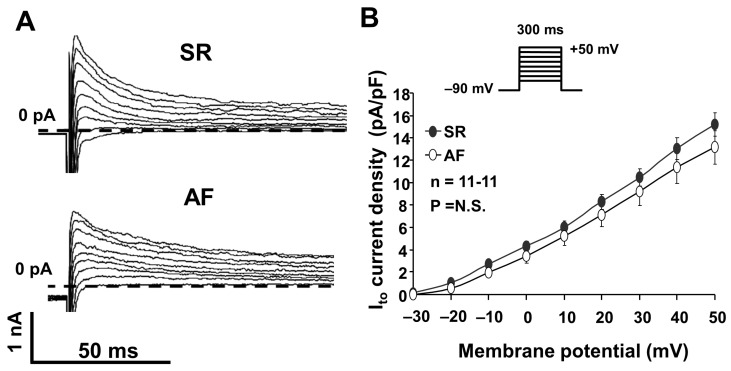
(**A**) Original transient outward potassium (I_to_) current registered in atrial myocytes isolated from dogs in sinus rhythm (SR) and atrial fibrillation (AF). The current was activated at a pulse frequency of 0.33 Hz by rectangular-shaped depolarizing voltage pulses of a 300 ms duration, ranging from −20 mV to 50 mV. (**B**) I_to_ current–voltage (I–V) characteristics in atrial cells isolated from SR and AF dogs (n = 11-11, *p* = N.S.).

The I_to_ in atrial myocytes was measured by applying two different patch-clamp protocols in isolated right atrial myocytes from dogs in SR and AF. First, we applied an action potential voltage command (see the inset of Figure 1A) to single atrial myocytes in a whole-cell configuration, and we identified I_to_ as a 3 mM 4 aminopyridine (4-AP) sensitive current (Figure 1A). Then, we measured I_to_ activated by 300 ms rectangular depolarizing pulses between −20 mV and +50 mV from the −90 mV holding potential, with a pulse frequency of 0.33 Hz. The recorded I_to_ current had a relatively large amplitude (14–15 pA/pF) and could be completely blocked by 3 mM 4-AP (Figure 1B and Figure 2).

We compared the I_to_ measured in right atrial myocytes isolated from dog hearts in SR and AF. As Figure 2A,B show, we found that the I_to_ peak current was somewhat smaller in AF dogs; however, this reduction was not statistically significant (Figure 2B). 

**Figure 3:**
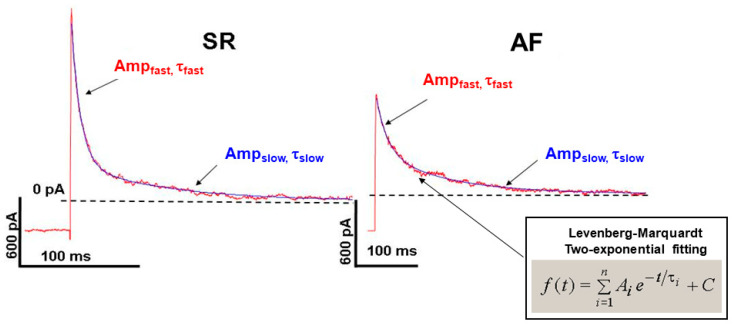
Kinetics of I_to_ current inactivation in isolated SR and AF canine atrial myocardial cells fitted with double exponential Levenberg–Marquardt equation.

We analyzed the I_to_ inactivation kinetics in atrial myocytes from dogs in SR and AF. The inactivation curve was best fitted by two exponential Levenberg–Marquardt functions. Accordingly, we identified a fast (τ_f_ ≈ 10–12 ms inactivation constant) and a slow (τ_s_ ≈ 120–150 ms inactivation constant) component of I_to_ (Figure 3). 

This slow component of the I_to_ kinetics in atrial myocytes was significantly slower than it was previously reported in left canine ventricular cells (τ_s_ ≈ 20–30 ms, measured at +20 mV) [11]. In the AF dogs, both of the fast and slow components of I_to_ were significantly decreased, compared with SR dogs (Figure 3, Table 1).

### 2.2. The Inward Rectifier Potassium Current (I_K1_)

**Figure 4:**
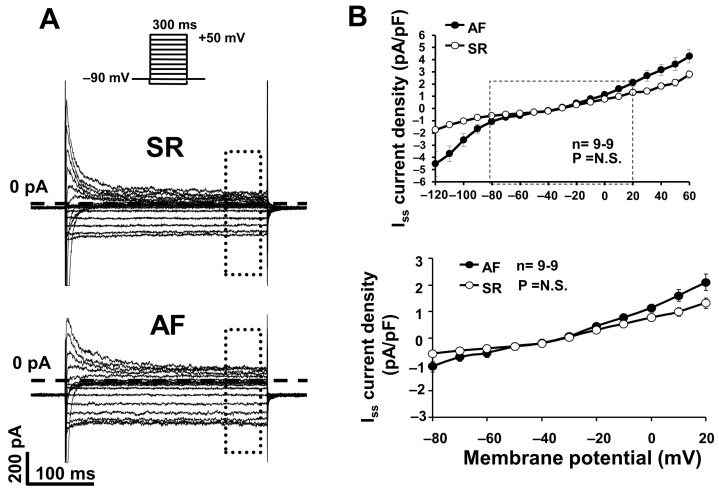
(**A**) Original inward rectifier potassium (I_K1_) current recordings in atrial myocytes isolated from dogs in sinus rhythm (SR) and atrial fibrillation (AF). I_K1_ current was measured as steady state current (I_ss_). (**B**) I_K1_ current–voltage (I–V) characteristics in atrial cells from dogs in SR and AF (n = 9-9; *p* = N.S.).

We measured the I_K1_ as a steady state current after 300 ms long depolarizing pulses from −140 mV to 60 mV, from the holding potential of −90 mV, at 0.33 Hz (I_ss_ current, Figure 4A,B). The current–voltage relationship demonstrates that the inward component of the I_K1_ is apparently upregulated in AF dogs; however, this enhancement was not statistically significant on the voltage range relevant to atrial AP (voltages between −80 and +20 mV) (Figure 4B, bottom panel).

### 2.3. The Acetylcholine-Sensitive Potassium Current (I_K,ACh_)

**Figure 5:**
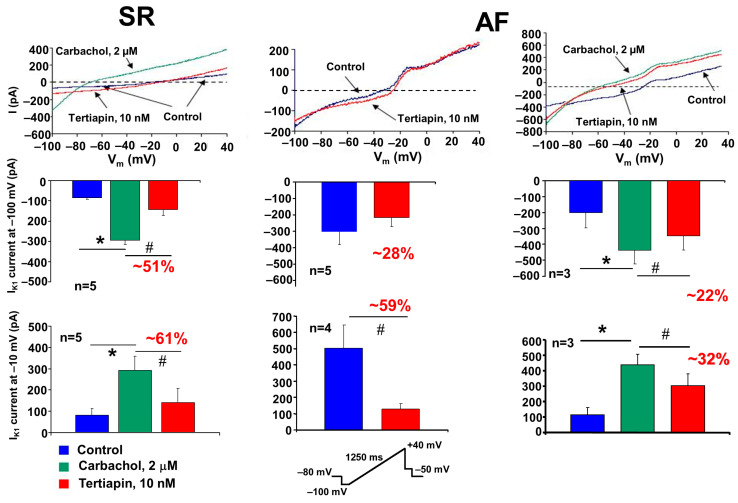
Acetylcholine-sensitive potassium (I_K,Ach_) currents in atrial myocytes isolated from dogs in sinus rhythm (SR) and atrial fibrillation (AF). The I_K,ACh_ current was activated by the cholinergic agonist carbachol (2 µM) using a ramp voltage protocol from −100 mV to 40 mV from the HP of −80 mV. The carbachol-induced I_K,ACh_ current was blocked with tertiapin (10 nM). Bar graphs represent the amplitude of I_K,ACh_ currents measured at −100 mV (inward range, middle row) and −10 mV (outward range, bottom row). The ramp protocol used is inset at the bottom of the figure. * denotes the statistical significance for carbachol measurements (between blue and green bars), while ^#^ denotes the statistical significance for tertiapin measurements (between blue and red bars or green and red bars, respectively).

The I_K,ACh_ was activated by the cholinergic agonist carbachol (2 µM) using a ramp voltage protocol. In SR dogs, in the absence of carbachol, we identified a small inward and outward current, which was largely increased after employment of 2 µM carbachol (Figure 5, upper panels) either at inward (measured at −100 mV membrane potential) or at outward (measured at −10 mV membrane potential) directions. 

This carbachol-activated current was significantly inhibited by the selective I_K,ACh_ current inhibitor, 10 nM tertiapin [12]. In AF dogs’ atrial myocytes, we could measure a large, constitutively active I_K,Ach_ current even in the absence of carbachol (Figure 5, upper middle panel). This current was also sensitive to tertiapin, since 10 nM tertiapin inhibited the constitutively I_K,ACh_ current either at inward or outward directions by 28% or 59%, respectively (Figure 5, bottom middle panel). These results are in agreement with previously described results, demonstrating that in atrial myocytes from chronic AF patients exists a tertiapin-sensitive constitutively active I_K,ACh_ current, which largely contributes to the electrophysiological atrial remodeling in AF [10]. 

Additionally, after treatment of atrial myocytes from AF dog cells with 2 µM carbachol, we recorded beside this constitutively active I_K,Ach_ current a large ligand-dependent and tertiapin-sensitive I_K,ACh_ current (Figure 5, left panels).

## 3. Discussion

### 3.1. The Transient Outward Potassium Current (I_to_)

Our experimental results clearly demonstrate the presence of a transient outward potassium current with a significant amplitude (about 14–15 pA/pF) in atrial myocytes isolated from control dogs (in SR). The presence of this current also causes the spike and dome configuration (phase 1, or early repolarization), which is highly characteristic of the atrial cell action potential (see Figure 1; SR atrial cell action potential). We measured the I_to_ currents of similar magnitude in myocytes isolated from the hearts of experimentally induced AF dogs (Figure 2A,B), which means that the peak of the I_to_ in our AF dog model was unaltered compared to SR. This result is controversial to previous data obtained from human AF, where the I_to_ current downregulation, the I_to_ protein subunits, and also mRNA downregulation were described [13,14,15]. The reason for this difference (human vs. dog) is not fully understood. In a similar 6-week tachypaced AF dog model study, the pore-forming subunit Kv4.3 mRNA downregulation was recorded; however, the native current was not investigated [7]. It is known that the I_to_ channel is a protein complex in dogs and consists of several α-subunits (Kv4.2, Kv4.3 and Kv1.4) and also β-subunits (KChIP2, miRP1, etc.). It is also known that the I_to_ complex in dogs is somewhat different than in humans (where Kv4.3 is the dominant α-subunit) [15], which may complement a damaged channel subunit function, i.e., this discrepancy could be a species difference between dogs and humans. However, we can also not exclude the fact that the applied 6-week tachypacing (400/min) protocol was not sufficient to reproduce the “downregulation” of the I_to_ current reported in human chronic or permanent AF [14]. 

We found that the I_to_ inactivation kinetics in atrial myocytes has two components, a fast one (τ~10–12 ms) and a slower (τ~122–130 ms) component (Figure 3 and Table 1). When comparing these atrial I_to_ data with those reported in dog ventricular myocytes, we found that the fast component of the current is similar (τ~4–5 ms), but the slow component from ventricular myocytes (τ~20–25 ms) is significantly slower in atrial myocytes [11]. These results may indicate that the I_to_ in atrial myocytes is not only involved in the phase 1 repolarization (the spike and dome configuration), but the slow component of the current may also contribute to the late (phase 3) repolarization at 100–150 ms. 

In AF dogs, I_to_ inactivation could also be best fitted with two exponential functions, but both faster and slower components were significantly decreased (on average, τ_f_ decreased from 11.7 ms to 17.8 ms, and τ_s_ from 122 ms to 180 ms), which may substantially contribute to atrial AP remodeling in AF.

### 3.2. The Inward Rectifier Potassium Current (I_K1_)

I_K1_ has a major role in the terminal repolarization and in the maintenance of the resting membrane potential [16]; however, more recent studies also described that I_K1_ also has a large outward component at around membrane voltages of −60 mV, and accordingly, may play a significant role in the phase 3 late repolarization [17]. Hereby, we reported the clear presence of the I_K1_ current in SR and AF dog atrial myocytes, and we showed that the I_K1_ was upregulated in AF, either at inward or outward directions (Figure 4A,B). This observation is in accordance with previous observations in chronic human AF samples, where the upregulation of both the native I_K1_ current and the Kir2.1 channel determining mRNA upregulation was described [9]. However, we analyzed in more detail the magnitude of the I_K1_ current on the voltage ranges relevant to dog cardiac potential (membrane voltages between −80 mV and +20 mV) and found that the I_K1_ current in AF dogs was not different from those measured in SR dogs, indicating that I_K1_ upregulation may not contribute to the electrical remodeling in this AF dog model (Figure 4B, bottom panel).

### 3.3. Acetylcholine-Activated Potassium Current (I_K,ACh_)

I_K,ACh_ is specially expressed only in atrial myocytes, and the activity is under the control of vagus nervus activation [18]. Released acetylcholine activates muscarinergic receptors and these receptors activate the I_K,ACh_ current, i.e., in normal conditions, without receptorial activation, the channel is inactive [19]. It is well known that the extrinsic and intrinsic autonomic (autonomic, sympathetic/parasympathetic) nervous system is a crucial contributor to the development and/or maintenance of AF. We know that activation of the vagus nerve shortens the atrial APD and ERP and that the dispersion/inhomogeneity of atrial repolarization is (also) increased due to the unequal release of acetylcholine [20]. It was also shown that the parasympathetic nervous system activation could be the base of the re-entry (rotors) arrhythmias and the AF formation [21]. The latter is frequently seen in clinical practice in the so-called vagotonic or “Coumel-type” AF [22]. 

Of course, the question arises whether changes in the intensity of the I_K,ACh_ current may also play a role in the development of Coumel-type AF (AF induced by vagus excitation, sleep, postprandial state, etc.). We know that vagal stimulation increases the I_K,ACh_ intensity and that current enhancement-induced remodeling also plays a crucial role in AF development. Dobrev et al. in 2001 described that, as a result, the atrial tachycardia-induced remodeling (ATR) upregulated I_K1_ upregulation, while the I_K,ACh_ underlying protein subunit GIRK4 expression was downregulated [9]. Later, the same group showed that in permanent AF, as a result of a phosphorylation-signaling mechanism, without any prior ligand stimulation, the I_K,ACh_ channels remain “constitutively” open [10]. This was a surprising result because it was the first study to demonstrate that the I_K,ACh_ channels without prior ligand stimulation are capable of active current transfer in specific arrhythmias (e.g., permanent AF).

This constitutively active form of I_K,ACh_ contributes to the development of triangular AP morphology in AF and the arrhythmogenesis of the re-entry type of arrhythmias [10]. The atrial-specific I_K,ACh_ inhibition (with tertiapin) does not lengthen the ventricular repolarization, indicating that it lacks proarrhythmic side effects (*Torsades de Pointes* ventricular tachycardia) unlike classical I_Kr_ inhibitors. Therefore, it is feasible that the selective inhibitors of the constitutively active I_K,ACh_ could be promising antiarrhythmic compounds in the therapy of parasympathetic activation-induced atrial fibrillation [10,23].

In our present experiments, we have confirmed the presence of a constitutively active I_K,ACh_ current (as shown in the middle panels of Figure 5). Accordingly, in AF cardiomyocytes, a tertiapin-sensitive current component can be identified even in the absence of the cholinergic agonist carbachol (red curve and bars), both at inward (at −100 mV) and outward (at −10 mV) directions. This demonstrates that the ligand-independent (even in the absence of acetylcholine) type of I_K,ACh_ current is also “constitutively” present in atrial myocytes isolated from tachypaced AF dogs. It should be added that this current is of very low density, especially in the outward direction, so it is questionable whether the constitutive I_K,ACh_ current is “strong” enough to induce by itself the electrical remodeling (marked atrial ERP and APD shortening) characteristic of AF [6].

Based on our present experiments, it seems reasonable to outline a new hypothesis. The parasympathetic (basic) tone is always present under normal conditions. Thus, the I_K,ACh_ current stimulated by vagus fibers/acetylcholine is continuously and always present in atrial myocytes, to which the constitutively activated I_K,ACh_ current may be added. This combined “atypical” ionic current (vagally induced and constitutively active I_K,ACh_ currents) may play a role in the recurring persistent or permanent forms of AF. Indeed, the experiments shown in the right panel of Figure 5 demonstrate that when myocytes isolated from dogs with atrial remodeling were further activated with carbachol, the generated current (which includes both constitutive and carbachol-induced I_K,ACh_ currents) is substantially larger (especially in the outward direction) in comparison with the carbachol-activated myocytes from SR dogs (Figure 5, lower right panel). We may conclude that this “combined current” may be sufficiently large to contribute to the triangularization and abbreviation of APD observed in AF remodeling [6].

To confirm this hypothesis, Juhász et al. recently showed that tertiapin was able to prevent experimental AF in dogs induced by rapid atrial cardiac stimulation in the setting of atrial electrical remodeling [24]. Further human studies are needed to confirm whether I_K,ACh_ inhibition will be useful in the prevention, elimination and/or prophylaxis of AF in clinical settings.

## 4. Materials and Methods

The experiments were performed on atrial myocytes isolated from dogs in SR and the experimentally induced tachypaced model of AF. Electrophysiological experiments were performed in atrial myocytes isolated from Beagle dogs’ hearts. The experimental data were collected between 2008 and 2022.

The experiments complied with the Guide for the Care and Use of Laboratory Animals (USA NIH publication No. 85–23, revised 1996). The protocols had been approved by the Ethical Committee for the Protection of Animals in Research of the University of Szeged, Szeged, Hungary (I-74-5-2012 and I-74-24-2017), and by the Department of Animal Health and Food Control of the Ministry of Agriculture (XIII/1211/2012 and XIII/3331/2017), and conformed to the rules and principles of the 2010/63/EU Directive. 

### 4.1. Atrial Tachypacing-Induced AF in Conscious Dogs

The tachypaced dog AF model was carried out by a method adapted from two groups [23,25] and has been previously described [24]. The Beagle dogs (weighing 12–13 kg) were acclimated to the experimental staff and equipment for one week before the start of the investigation. Pacemaker and pacemaker electrode implantation procedures were performed following ketamine anesthesia (Richter Gedeon Ltd., Hungary; induction: 10 mg/kg, i.v., maintenance: 2 mg/kg, every 20 min) and xylazine (CP-Pharma Handelsges, Germany; induction: 1 mg/kg, maintenance: 0.2 mg/kg, every 20 min, Baczkó et al. 2014). Two bipolar pacemaker electrodes (Synox SX 53-JBP and Synox SX 60/15-BP; Biotronik Hungary Kft, Budapest, Hungary) were placed in the right atrial appendage and the apex of the right ventricle, respectively, and the electrodes were connected to pacemakers (Logos DS and Philos S; Biotronik Hungary Kft., Budapest, Hungary) placed in subcutaneous pockets in the neck region, followed by radiofrequency catheter ablation of the AV node. The pacemakers were programmed using the ICS 3000 programmer (Biotronik Hungary Kft., Budapest, Hungary). After recovery from surgery (3–5 days), right atrial tachypacing was started at a rate of 400 beats/min, which was maintained for at least 6–7 weeks before the experiments to allow for electrical remodeling of the atria (monitored by measuring the right atrial effective refractory period (AERP) every other day). The AERP was measured at baseline cycle lengths (BCLs) of 150 and 300 ms for 10 stimulations (S1) and one extrastimulus (S2).

### 4.2. Isolation of Right Atrial Myocytes

Before heart removal, the animals received 400 U/kg Na-heparin i.v. treatment. After sedation (xylasin 1 mg/kg i.v. and ketamine 10 mg/kg i.v.) and anesthesia (pentobarbital, Sigma Chemical, St. Louis, MO, USA, 30 mg/kg, i.v.), the heart was rapidly removed by lateral thoracotomy and washed by physiological nutrient solution at 4 °C. The right atrium was perfused by 1 mL heparin and a 1 mM CaCl_2_-supplemented isolation solution (NaCl 135, KCl 4.7, KH_2_PO_4_ 1.2, MgSO_4_ 1.2, HEPES 10, glucose 10, taurine 20, NaHCO_3_ 4.4, Na-pyruvate 5 (mM/L) and pH 7.2 NaOH) in a Langendorff perfusion apparatus through a cannula which was set into the coronaria. After the blood was washed out (5 min), a Ca-free isolation solution was applied for 10 min. Then, we started the enzymatic digestion in the Ca-free isolation solution with Collagenase (Clostridium histolyticum type I, 0.54 mg/mL Sigma Chemical, St. Louis, MO, USA), 0.1% BSA-t (bovine serum albumin fraction V; Sigma Chemical) and in the 15th minute, we added protease (type XIV, 0.05 mg/mL; Sigma Chemical). After 40 min, the right atrium was cut into small pieces and replaced into a 1 mM CaCl_2_- and 1% BSA-containing isolation solution for 15 min at 37 °C. After trituration and filtration, we obtained the single atrial cell suspension, which was washed twice by a fresh 1 mM CaCl_2_ isolation solution. The atrial myocyte-containing solution was kept for experiments at room temperature.

### 4.3. Electrophysiological Measurements

The atrial myocytes were placed into a cell bath fixed to an Olympus IX51 inverted microscope, Tokyo, Japan and superfused with normal Tyrode’s solution at 37 °C (NaCl 144, KCl 4, NaH_2_PO_4_ 0.4, MgSO_4_ 0.53, HEPES 5, glucose 5.5, CaCl_2_ 1.8, (mM/L) and pH 7.4 NaOH). Patch-clamp pipettes having 2.0–2.5 MΩ resistance were filled by the following pipette solution: K-aspartate 100, KCl 45, MgATP 3, MgCl_2_ 1, EGTA 10, HEPES 5 (mM/L) and pH: 7.2 KOH. The transmembrane currents were registered in whole-cell configurations at 37 °C by an Axopatch 200B patch-clamp amplifier, under Digidata 1440 analog–digital converter and Axon pClamp 10.3 software control (Molecular Devices; Union-City, CA, USA). For the analysis, we used Axon pClamp 10.3 software. We used the following ion channel blockers: nisoldipine, 1 µM (I_CaL_ inhibitor); dofetilide, 0.1 µM (I_Kr_ inhibitor); HMR-1556, 0.5 µM (I_Ks_ inhibitor); tertiapin 10 nM (I_K,ACh_ inhibitor); and 4-aminopyridine, 3 µM (4-AP for I_to_ inhibition) [26].

### 4.4. Statistics

The results are expressed as mean ± SEM. The normality of distributions was verified using the Shapiro–Wilk test, and the homogeneity of variances was verified using Bartlett’s test in each treatment group. When comparing values between SR and AF myocytes (Figure 2, Figure 3 and Figure 4 and Table 1), statistical comparisons were made using an analysis of variance (ANOVA) for repeated measurements, followed by Bonferroni’s post-hoc test. 

We applied Student’s *t*-test for paired data when measuring the effect of carbachol and/or tertiapin on the I_K,ACh_ current (Figure 5) in self-controlled patch-clamp experiments. 

Differences were considered significant when *, ^#^ *p* < 0.05, accordingly.

## 5. Conclusions

Due to its slow inactivation kinetics, the atrial I_to_ current may contribute to late repolarization in atrial myocytes to a greater extent than has been shown in ventricular myocytes. The I_to_ current in our dog model, in contrast to human data, was only slightly “downregulated” in AF, but its inactivation kinetics were significantly slowed.

The presence of the constitutively active I_K,ACh_ demonstrates that atrial electrical remodeling has occurred in our AF dog model. Together, ligand-dependent and constitutively active I_K,ACh_ currents presumably play an important role (at least in dogs) in the development of the triangularized and abbreviated shortened action potential waveform characteristic of AF.

Based on the results of the present study, we hypothesized that the parasympathetic activation-induced I_K,ACh_ is present in atrial myocytes even under normal conditions, and this current can be supplemented with a constitutively active I_K,ACh_. This later “atypical” current can contribute to the development of the recurring (persistent or permanent) forms of AF. The study of Juhász et al. [24] supported this assumption by demonstrating that tertiapin was protective against rapid pacing-induced atrial remodeling. Further studies are required to examine the potential role of I_K,ACh_ inhibition under clinical conditions to prevent and/or treat atrial fibrillation.

## Figures and Tables

**Table 1:** Inactivation time constants (τ) and the density values of the corresponding current amplitudes (Dens_Amp_) of the fast and slow components of the I_to_ current measured at 20 mV in isolated atrial myocytes from dogs in SR and AF.

Parameter	τ_fast_ (ms)	τ_slow_ (ms)	Dens_Ampfast_ (pA/pF)	Dens_Ampslow_ (pA/pF)
SR (n = 10)	11.70 ± 0.76	121.9 ± 8.82	5.80 ± 0.61	2.32 ± 0.38
AF (n = 11)	17.83 ± 2.97 *	179.2 ± 25.42 *	4.22 ± 0.7	2.71 ± 0.35

* denotes statistical differences between SR and AF groups. * *p* < 0.05.

## Data Availability

Data is contained within the article.

## List of Abbreviations

AF	atrial fibrillation
4-AP	4-aminopyridine
AP	action potential
APD	action potential duration
ATR	atrial tachycardia-induced remodeling
CCh	carbachol
ERP	effective refractory period
ES	extra systole
HP	holding potential
I_CaL_	L-type calcium current
I_K,ACh_	acetylcholine-sensitive potassium current
I_K1_	inward rectifier potassium current
I_to_	transient outward potassium current
SR	sinus rhythm
